# Comparison of Corneal Epithelial Thickness Map Measured by Spectral Domain Optical Coherence Tomography in Healthy, Subclinical and Early Keratoconus Subjects

**Published:** 2019

**Authors:** Farshad OSTADIAN, Fereydoun FARRAHI, Atefeh MAHDIAN RAD

**Affiliations:** 1 Department of Ophthalmology, Faculty of Medicine, Infectious Ophthalmic Research Center, Ahvaz Jundishapur University of Medical Sciences Ahvaz, Iran

**Keywords:** Epithelial Thickness Map, Keratoconus, Subclinical Keratoconus, Early Keratoconus, Spectral Domain Optical Coherence Tomography, SD-OCT

## Abstract

The aim of this study was to compare epithelial thickness map obtained by Spectral Domain Optical Coherence Tomography (SD-OCT) of eyes with myopic astigmatism but without keratoconus, subclinical and early keratoconus. Sixty-three eyes were divided into three groups; myopic astigmatism without keratoconus, subclinical and early keratoconus. Corneal epithelial thickness map was obtained by SD-OCT for all patients and compared between the 3 groups. Mean ± Standard Deviation of epithelial thickness in the area of minimum corneal epithelial thickness, in the one eighth part of the inferior (I) and in the one eighth part of the temporal (T) were 56.64±2.82 µm, 59.00±3.24 µm and 60.40±4.93 µm respectively in subclinical group. Three parameters on epithelial maps obtained by SD-OCT was significantly different in the 2 groups: I and T corneal epithelial thickness map was thicker in subclinical keratoconus (P<0.02 and P<0.02 respectively). Epithelial map uniformity indices were different between the groups, as Superior-I, Superonasal-Inferotemporal were lower (P<0.00 and P< 0.01 respectively) but T-nasal was higher in the subclinical group (P<0.02). The area with minimum epithelial thickness had a significantly lower amount in early keratoconus group compared to the other two groups (P<0.00). In conclusion, corneal epithelial thickness map provided early detection of keratoconus in the subclinical stage with compensatory epithelial thickening of inferior and one eighth of temporal compared to total corneal thickness and changes in epithelial map uniformity indices may lead to early detection of subclinical keratoconus from normal cornea.

## INTRODUCTION

Keratoconus is a common disease which affects one per 2000 individuals [1]. It is a non-inflammatory and progressive disease which affects both eyes bilaterally and symmetrically. In this disease, the central or paracentral cornea is protuberated and progressively thinned and ultimately becomes cone-shaped [1]. Unrecognized early stages of keratoconus not only leads to iatrogenic corneal ectasia following laser keratorefractive surgery [2], but also deprives patients with keratoconus from early therapeutic interventions such as corneal collagen cross-linking [3]. So early diagnosis of keratoconus is still an important challenge for refractive surgeons [4, 5]. The gold standard diagnostic method for ectatic corneal disorders, such as pellucid marginal degeneration and keratoconus is corneal topography [5-7], which shows normal, suspicious and abnormal corneas. However, based on topography and without additional risk factors, there are reports of cases of ectasia following LASIK in healthy eyes, indicating inadequacy of topography to distinguish early stages of keratoconus [8-11]. 

The epithelium is the superficial layer of the cornea and considering its uniform coating, plays an essential role in maintaining the optical quality of the eye [12]. This layer can alter and rebuild itself in response to irregularities and changes in the stroma [13, 14], and ultimately maintain the smoothness and uniformity of the anterior surface of the eye, which is the basis for evaluation in the topography. This fact could justify lack of keratoconus diagnosis in early stages using topography [4, 5, 12,]. 

There are several ways to measure the thickness of the epithelium in different parts of the cornea, including confocal imaging [16], very high-frequency ultrasound corneal analysis [15, 17] and Spectral Domain Optical Coherence Tomography (SD-OCT) [18]. Because the immersed probe in the fluid needs to be in contact with the eye, ultrasonography may not be safe and is usually not considered [12]. But the OCT, which is a non-contact and reliable method, can remarkably delaminate the surface of the cornea and accurately demonstrate the thickness pattern of the corneal epithelium, due to its high axial resolution [18]. In this study, we aimed to measure the corneal epithelium thickness with the help of SD-OCT in healthy persons, patients with subclinical stages and those with early stages of clinical keratoconus. Comparison of these parameters may help to find a way to differentiate normal, suspected and keratoconic eyes.

## METHODS

This retrospective study was conducted from November 2017 to November 2018 on patient population requesting refractive surgery in Imam Khomeini Hospital (affiliated to Ahvaz Jundishapur University of Medical Sciences). The study protocol was approved by Ahvaz Jundishapur University of Medical Sciences Ethics Committee (approval number IORC-9707). The study was conducted in accordance with the Declaration of Helsinki. The inclusion criteria were ≥ 18-year-old patients with refractive error and requesting refractive surgery, having spherical myopia less than -6 diopters (D), astigmatism less than -4D for normal group participants, myopia and astigmatism (spherical equivalent) of less than -8D in the keratoconus group participants. Exclusion criteria were corneal hydrops, history of any eye surgery, corneal scar, contact lens wearing, keratometry of greater than 53D, history of any episodes of corneal edema, pregnancy or lactation and any systemic disease e.g. diabetes mellitus and Marfan syndrome. Considering the inclusion and exclusion criteria, 63 patients were included in this study. A written informed consent was obtained from all participants following detailed explanation of the study. Age, sex, and personal and familial history of the ophthalmological issues were registered. All participants underwent full eye examination including uncorrected and best corrected visual acuity (BCVA) measurement using the Snellen chart, slit lamp biomicroscopy, and retinal examination with a +90D non-contact lens as well as intraocular pressure measurement using the Goldman applanation tonometer. For all patients corneal scheimpflug imaging with Pentacam (Oculus, Lynnwood, WA, The USA) and corneal epithelial thickness mapping with SD-OCT (Spectralis Heidelberg Engineering; Germany) at a certain time (from 10 am to 2 pm and at least 2 hours after awakening) and by the same operator were obtained. 

Normal quantitative parameters and patterns in Pentacam, along with normal vision was characterized as normal (myopic astigmatism) group. People with normal vision but suspicious findings on Pentacam were characterized as subclinical group. Patients with BCVA≤ 20/25 and at least one keratoconus criterion in the topographic maps including, asymmetric bow tie pattern with a skewed radial axis, steep central of inferior zone or claw-shaped pattern, slit lamp keratoconus signs e.g. apical thinning, Vogt's striae, Fleischer's ring, Rizutti sign, Munson's sign, in the presence of abnormal findings in Pentacam investigation were considered as early stages of keratoconus group )based on the Amsler-krumeich scale) [19]. We used mean and standard deviations (SD) for describing quantitative parameters and frequency and percentage for describing qualitative variables. Data analysis was performed by Pearson correlation coefficient, Mann-Whitney test and one-way ANOVA. Regression methods were used if necessary. P lesser than 0.05 was considered statistically significant. The analyses were performed using version 20, SPSS software (IBM Corp., Armonk, NY, USA).

## RESULTS

Of 63 participants, 24 patients were in the normal group, 17 in the subclinical group and 22 in early stages of keratoconus group. Patients’ BCVA in normal and sub-clinical keratoconus groups were almost similar, but in the early keratoconus BCVA was lower compared to the other groups. There was no significant difference between the control and subclinical groups regarding the spherical equivalent (SE) (P = 0.28), but it was significantly more in the keratoconus group compared to the subclinical group (P = 0.04). Refractive error of the keratoconus and the control group did not have a significant difference (P = 0.54). The cylinder power was almost similar between the control and subclinical groups (P = 0.18), but it was more in the keratoconus group compared to both subclinical (P = 0.01) and control groups (P = 0.00) (Table 1). The results of the steep K and flat K in the center of the cornea were almost similar between subclinical and control groups (P = 0.66) and (P = 0.24) respectively, but significantly higher in the keratoconus group compared to both subclinical (P = 0.00) and (P = 0.05) respectively and control groups; (P = 0.00) and (P = 0.00) respectively. The posterior corneal elevation was significantly higher in the subclinical keratoconus group compared to the control group (P = 0.04). Also the posterior corneal elevation was higher in the keratoconus group compared to both subclinical (P = 0.00) and control groups (P = 0.00). Anterior corneal elevation was not significantly different between subclinical and control groups (P = 0.33) but was significantly more in the keratoconus group compared to both subclinical (P = 0.00) and control groups (P = 0.00). There was no significant difference between subclinical and control groups regarding the Q value (P = 0.33), but it was significantly higher in the keratoconus group compared to both subclinical (P = 0.00) and control groups (P = 0.00). The BAD-D (The Belin/Ambrósio enhanced ectasia display final 'D' index) did not have any significant difference between subclinical and control groups (P = 0.12), but it was higher in the keratoconus group compared to both subclinical (P = 0.00) and control groups (P = 0.00) (Table 1). Thickness of the thinnest point of the cornea was different in all the three groups (P = 0.00) so that it was thinner in the subclinical group (P = 0.00) and thinnest in the keratoconus group (P = 0.00). The vertical coordination of this point (ANOVA: 0.76) and the difference in the thickness of the superior and inferior parts measured by Pentacam (ANOVA: 0.97) did not have a statistically significant difference between the three groups. Although the progression index difference between the control and subclinical groups was not statistically significant (P = 0.42), progression index was significantly more in the keratoconus group compared to both subclinical (P = 0.00) and control groups (P = 0.00) (Table 2). Total corneal thickness in temporal and minimum regions are significantly thinner in the keratoconus group compared to the subclinical group; (P = 0.02) and (P = 0.01) respectively (Table 3). Interestingly, the mean of the epithelial thickness of the central, superior, inferior, nasal and temporal regions in the subclinical group is approximately 3-4 microns more than the control and keratoconus groups, and this increase in thickness at the inferior area (P = 0.03) and (P = 0.02) respectively and temporal area (P = 0.01) and (P = 0.02) respectively is statistically significant. Nonetheless, the thickness of the epithelium is similar between the keratoconus and control groups (P = 0.92) and (P = 0.71) at the inferior and temporal area respectively. The minimum thickness of the epithelium is also different between the three groups, but this difference is only significant between the subclinical group and keratoconus group (P = 0.00). Another critical point of epithelial thickness results was the increase in SD in the keratoconus group, which confirms an irregular increase in the epithelial level in the keratoconus group (Table 3).

**Table 1:** Slit Lamp Examination and Pentacam Results in Three Study Groups

Group				P-value			
Parameters	Early keratoconus	Subclinical keratoconus	Control	control vs early keratoconus	Subclinical vs early keratoconus	Control vs Subclinical keratoconus	ANOVA
BCVA (log MAR)(Mean ± SD)	0.25±0.27	0.02±0.05	0.03±0.07	**0.00**	**0.00**	0.99	**0.00**
Spherical Equivalent(D) Mean ± SD	-4.84±2.52	-3.0±2.24	-4.12±1.83	0.54	**0.04**	0.28	**0.04**
Cylinder(D) Mean ± SD	-3.83±2.10	-2.10±1.66	-1.10±1.00	**0.00**	**0.01**	0.18	**0.00**
Steep meridian (degree) Mean ± SD	95.35±31.94	83.23±13.22	87.84±23.79	0.61	0.34	0.85	0.32
Steep K (D) Mean ± SD	48.07±3.47	44.64±1.90	45.35±1.27	**0.00**	**0.00**	0.66	**0.00**
Flat K (D) Mean ± SD	42.92±2.21	44.24±1.63	46.11±2.83	**0.00**	**0.05**	0.24	0.06
I-S diff(D) Mean ± SD	6.93±6.23	1.35±1.98	0.07±0.60	**0.00**	**0.00**	0.58	**0.00**
Post elevation (µm) Mean ± SD	50.82±14.91	19.41±9.18	11.04±3.88	**0.00**	**0.00**	**0.04**	**0.00**
Ant elevation (µm) Mean ± SD	23.14±9.35	8.29±4.96	5.25±3.11	**0.00**	**0.00**	0.33	**0.00**
Q value (Mean ± SD)	-0.76±0.31	-0.40±0.14	-0.32±0.12	**0.00**	**0.00**	0.49	**0.00**
BAD-D (Mean ± SD)	7.31±2.56	2.35±1.05	1.26±0.71	**0.00**	**0.00**	0.12	**0.00**

µm: Micrometer; SD: Standard Deviation; D: Diopter; I-S: Inferior-Superior; K: Keratometry reading; BAD-D: The Belin/Ambrósio enhanced ectasia display final 'D' index; BCVA: Best Corrected Visual Acuity; log MAR: Logarithm of the Minimum Angle of Resolution; VS: versus; ANOVA: Analysis of variance.

**Table 2:** Corneal Parameters Measured With Pentacam in Three Study Groups

Group				P-value			
Parameters	Control	Subclinical keratosis	Early keratoconus	ANOVA	Control vs Subclinical keratoconus	Subclinical vs early keratoconus	control vs early keratoconus
Thinnest location (µm) (Mean±SD)	522.46±27.75	480.24±38.20	443.18±26.78	**0.00**	**0.00**	**0.00**	**0.00**
Coordination (Mean±SD)	-405.0±292.13	-481.76±418.18	-426.09±294.89	0.76	0.77	0.87	0.98
Superior-inferior difference (µm) (Mean±SD)	23.0 0±14.31	22.94±14.85	23.91±20.49	0.97	1.00	0.98	0.98
Progression index, (Mean±SD)	1.02±0.17	1.13±0.23	1.96±0.36	**0.00**	0.42	**0.00**	**0.00**

µm: Micrometer; SD: Standard Deviation; VS: versus; ANOVA: Analysis of variance.

The epithelial map uniformity indices superior and inferior (Sup-Inf), supranasal and infratemporal (SN-IT) epithelial thickness difference showed a significant decrease in subclinical group compared to both the keratoconus (P = 0.00) and (P = 0.00) respectively and control groups (P = 0.00) and (P = 0.01) respectively and temporal and nasal (T-N) epithelial thickness difference was significantly higher in subclinical group compared to the normal group (P = 0.02). In the keratoconus group, the difference of max-min, Sup-Inf, T-N, SN-IT in the corneal thickness was significantly higher than the other two groups (P = 0.00 for all), but the difference of ST-IN in the corneal thickness did not vary significantly compared to both subclinical (P = 0.20) and control groups (P = 0.60) (Table 4).

**Table 3:** Details of the Corneal Parameters Measured Using SD-OCT in Three Study Groups

Groups				P-value			
	control	Subclinical keratoconus	Early keratoconus	ANOVA	Control vs Subclinical keratoconus	Subclinical vs early keratoconus	control vs early keratoconus
Corneal thickness (µm) Mean±SD							
**central**	518.96±27.88	485.18±38.36	465.41±33.92	**0.00**	**0.01**	0.19	**0.00**
**minimum**	503.00±28.59	464.59±39.22	429.86±28.56	**0.00**	**0.00**	**0.01**	**0.00**
**maximum**	566.42±28.35	533.12±39.95	526.59±27.84	**0.00**	**0.01**	0.82	**0.00**
**Superior**	536.29±27.93	505.05±40.75	499.50±21.55	**0.00**	**0.00**	0.84	**0.00**
**Inferior**	524.83±27.09	488.41±41.35	476.54±25.20	**0.00**	**0.00**	0.49	**0.00**
**Temporal**	518.50±29.08	484.47±38.53	455.90±28.98	**0.00**	**0.00**	**0.02**	**0.00**
**Nasal**	533.75±27.08	499.58±41.75	497.54±23.78	**0.00**	**0.00**	0.97	**0.00**
Epithelial thickness (µm) mean±SD							
**Central**	57.67±4.21	59.82±3.52	57.00±5.77	0.16	0.35	0.18	0.89
**Minimum**	55.04±4.32	56.64±2.82	52.18±5.7	**0.01**	0.27	**0.00**	0.38
**Maximum**	60.96±5.66	63.53±5.93	62.32±7.95	0.47	0.48	0.85	0.79
**Superior**	56.83±4.30	59.17±3.24	56.40±6.16	0.17	0.31	0.21	0.95
**Inferior**	56.20±4.27	59.00±3.24	56.06±7.11	**0.04**	**0.03**	**0.02**	0.92
**Temporal**	56.11±4.35	60.40±4.93	56.68±6.05	**0.02**	**0.01**	**0.02**	0.71
**Nasal**	56.33±4.26	59.70±4.66	56.31±5.76	0.06	0.10	0.11	1.00

SD-OCT: Spectral Domain Optical Coherence Tomography; µm: Micrometer; SD: Standard Deviation; ANOVA: Analysis of variance.

**Table 4:** Thickness Difference of Corresponding Areas of the Cornea Using SD-OCT in Three Study Groups

Groups				P-value			
Corneal Thickness (µm) Mean±SD	Control	Sub clinical keratoconus	Early keratoconus	ANOVA	Control vs Subclinical keratoconus	Subclinical vs early keratoconus	control vs early keratoconus
**Max-Min**	66.17±14.81	68.53±12.30	95.18±28.03	**<0.000**	0.93	**0.00**	**0.00**
**Sup-Inf**	11.04±8.06	16.53±9.84	27.68±15.65	**<0.000**	0.34	**0.02**	**0.00**
**T-N**	(-16.08)±(8.90)	(-18.94)±(7.38)	(-43.27)±(18.72)	**<0.000**	0.79	**0.00**	**0.00**
**ST-IN**	(-3.83)±(10.23)	(-1.12)±(8.87)	(-9.86)±(13.76)	**0.049**	0.75	0.20	0.60
**SN-IT**	19.38±6.63	28.35±7.79	50.32±20.08	**<0.000**	0.11	**0.00**	**0.00**
Epithelial thickness (µm) Mean±SD							
**Max-Min**	5.88±5.57	6.88±6.40	9.40±5.2	0.109	0.25	0.39	0.11
**Sup-Inf**	0.52±0.81	(-0.72)±(0.39)	0.39±0.42	**<0.000**	**0.00**	**0.00**	0.46
**T-N**	(-0.21)±(0.58)	0.34 ± 0.89	0.31±0.77	**0.024**	**0.02**	0.92	**0.01**
**ST-IN**	0.17±1.17	0.06±0.43	0.14±0.71	0.924	0.93	0.96	0.99
**SN-IT**	(0.04)±(0.90)	(-0.65)±(0.82)	(0.13)±(0.99)	**0.020**	**0.01**	**0.00**	0.72

SD-OCT: Spectral Domain Optical Coherence Tomography; µm: Micrometer; SD: Standard Deviation; VS: versus; Max-Min: Maximum-Minimum; Sup-Inf: Superior- Inferior; T: Temporal; N: Nasal; ST: Superotemporal; IN: Inferonasal; SN: Superonasal; IT: Inferotemporal; ANOVA: Analysis of variance.

## DISCUSSION

We found that mean epithelial thickness in the area of minimum corneal epithelial thickness, in I and in T were 56.64±2.82 µm, 59.17±3.24 µm and 60.40±4.93 µm respectively in subclinical group. Three parameters on epithelial maps obtained by SD-OCT were significantly different in 2 groups: I and T corneal epithelial thickness map was thicker in subclinical keratoconus. Epithelial map uniformity indices were different between the groups, as Sup-Inf, SN-IT were significantly lower but T-N was significantly higher in the subclinical group. The area with minimum epithelial thickness had a significantly lower amount in early keratoconus group compared to the other two groups.

Keratoconus demonstrates a wide range of severity, and therefore, there are several approaches and treatment-based classifications for it. In this study, we followed the Amsler-krumeich scale [19]. The OCT corneal pachymetry mapping with its fast acquisition time seems promising for evaluating highly astigmatic corneas, as in early keratoconus [20]. At the apex of early keratoconus focal steepening would be compensated by corneal epithelial thinning [12, 15, 21, 22]. Thus focal epithelial thinning detection can be a more sensitive tool to identify very early stage of keratoconus [4]. In this study, we used non-contact high axial resolution SD-OCT for the corneal epithelial thickness mapping and measured the thickness at the central 5 mm due to high repeatability and capability in this region [20, 23] and because the cone of keratoconus is most often created in this region [24]. Our results showed that the mean values of epithelial thickness at the central 5 mm in the central, superior, inferior, nasal and temporal regions of the subclinical group were approximately 3-4 microns greater than the control and early keratoconus groups, and this increased thickness in the inferior and temporal areas of the subclinical group were statistically significant. However, the thickness of the epithelium in the keratoconus and the control groups are similar. The increase in the thickness of the epithelium in the lower region is significant, while this is not the case in the stroma and the entire cornea. This finding confirms the role of determining epithelial thickness in early detection of early stages of keratoconus. Unlike these two, the minimal epithelial thickness in the keratoconus group is lower than the subclinical group. Also, we found that stromal thickness map displayed thinning in the inferior and temporal regions, but only in the temporal region is statistically significant, while the thinning in the inferior region was compensated by increase in epithelial thickness. However, this thickening of epithelium was statistically significant. As a result, we could say that in the inferior area of the central 5 mm of the cornea, early epithelial changes, and the analysis of the corneal epithelial pattern may assist to diagnose ectatic corneal disorders earlier. We can distinguish normal group from subclinical keratoconus group by comparing thickness of total cornea. However, by comparing epithelial thickness, between subclinical keratoconus and early keratoconus, inferior region shows significant difference. This finding shows that using epithelial thickness is better than total corneal thickness to distinguish early keratoconus from subclinical keratoconus and is helpful for early treatment of early keratoconus. In subclinical keratoconus the corneal epithelium showed an epithelial doughnut pattern so that there is a localized central thinning and a ring of thick epithelium around it. In subclinical keratoconus the central epithelial thickness was thinner than normal eyes and this difference was statistically significant [5, 15]. We did not find the epithelial doughnut pattern reported in subclinical keratoconus by Reinstein et al [17] in our study. This difference may be due to non-uniformity of grouping in these studies.

Temstet C et al. showed that the epithelial thinning zone is in accordance with the area of sharpest corneal curvature and suggested that the diagnosis of epithelial thinning in the thinnest corneal region can be used to detect subclinical keratoconus cases, but it is not adequate as a single diagnostic criterion [25]; however, it does not correlate with our study. Nonetheless, it has been shown in various studies that the compensatory thickening of corneal epithelium masks the focal steepening in the corneal keratoconic apex [12, 13, 23]. Therefore, separate measurements of epithelial thickness and recording focal changes in the corneal epithelium could be considered as a criterion for the diagnosis of primary stages of keratoconus with more sensitivity [4]. Several researchers have reported that keratoconic corneas are thinner than normal ones [15, 25]. Haque et al. reported that in subclinical keratoconus epithelial thickness at the corneal center is thinner than normal eye and average of this difference is 4.7 µm [27]. Temstet C et al. showed that the location of the thinnest epithelial thickness in subclinical keratoconus is different from normal eye so that in subclinical keratoconus is located inferiorly, and this difference is statistically significant [25]. Reinstein et al. determined that in keratoconus, the thinnest point of corneal epithelium is located at the IT quadrant [15]. This is in contrast with our results, but this may be due to unmatched classification in the two studies. Li Y et al. showed that epithelial thickness at the corneal center in subclinical keratoconus and normal eyes has no statistically significant difference, but their study revealed difference between the S-I, min–max epithelial thickness [12]. In our study the epithelial map uniformity indices Sup-Inf, and SN-IT epithelial thickness difference showed a significant decrease in subclinical group compared to the two other groups and T-N epithelial thickness difference was significantly higher in subclinical group compared to the normal group. These data confirm and complete other information concerning epithelial thickness, as they all help to detect early keratoconus (while corneal and stromal thickness do not display asymmetry yet, the epithelium demonstrates it). Variables of OCT pachymetry are diagnostic in keratoconus screening [4, 12]. Ponce et al. measured and compared the thickness of the thinnest region of the cornea with three devices; Pentacam, anterior segment OCT and ultrasound biomicroscopy. They showed that OCT pachymetry map scans had higher accuracy and appropriate reliability, especially in post LASIK corneas [29]. This can increase the strength of the results with SD-OCT.

The strengths of our study include a careful evaluation of the epithelial thickness and its comparison with thickness of cornea and the site of corneal elevation in keratoconus and epithelial mapping of different areas of the cornea. Our study drawbacks are limitation of cases and groups to show clinical range of keratoconus, failure to observe epithelial changes over time and inability to show cut-off points to differentiate subclinical keratoconus from normal cornea. We suggest performing further studies with more cases and a wider grouping. Patients should also be monitored for longer periods of time so epithelial changes are more evident.

## CONCLUSIONS

In patients with subclinical keratoconus, the epithelial thickness increases to mask the thinning of stroma thus corneal epithelial map uniformity indices scans (S-I; SN-IT; T-N) could help early diagnosis of the subclinical keratoconus and differentiating them from normal eyes. Also epithelial thickening of the inferior area could help detection of early keratoconus from subclinical keratoconus.
